# Diamond Etching Beyond 10 *μ*m with Near-Zero Micromasking

**DOI:** 10.1038/s41598-019-51970-8

**Published:** 2019-10-30

**Authors:** Marie-Laure Hicks, Alexander C. Pakpour-Tabrizi, Richard B. Jackman

**Affiliations:** London Centre for Nanotechnology & Department of Electronic and Electrical Engineering, University College London, 17-19 Gordon Street, WC1H 0AH London, UK

**Keywords:** Electronic devices, Electronic devices

## Abstract

To exploit the exceptional properties of diamond, new high quality fabrication techniques are needed to produce high performing devices. Etching and patterning diamond to depths beyond one micron has proven challenging due to the hardness and chemical resistance of diamond. A new cyclic Ar/O_2_ - Ar/Cl_2_ ICP RIE process has been developed to address micromasking issues from the aluminium mask by optimising the proportion of O_2_ in the plasma and introducing a preferential “cleaning” step. High quality smooth features up to, but not limited to, 10.6 *μ*m were produced with an average etched surface roughness of 0.47 nm at a diamond etch rate of 45 nm/min and 16.9:1 selectivity.

## Introduction

Not only a gemstone, diamond is an exceptional engineering material^1–5^. High quality processing and fabrication techniques are crucial to progress from substrate material to high functioning device to apply the properties of diamond to biotechnology, quantum technology and electronics.

Diamond is a physically hard and chemically inert material; traditional fabrication methods used in the semiconductor industry have not been easily transferred to this material system for etching. Photoresists generally employed as masks in other semiconductor systems have insufficient lifetimes in the plasma to achieve significant etch depths in diamond, leaving metals or oxides as the main masking options^6–8^. These masking materials are however known to generate micromasking, defects produced by sputtered mask particles across the etched surface. Micromasking leads to the formation of pronounced ‘spikes’ protruding from the etched surface with clearly calamitous consequences for subsequent device fabrication^8–10^.

Up until recently there were no reports of deep etching of diamond (>5 *μ*m) and few reports of defect-free etched diamond surfaces. Progress has, however, been made for diamond deep etching using shadow masks and with a nickel mask/SF_6_ plasma process which both produced deep etched features with near-zero micromasking^11–13^. Toros *et al*. presented a multi-step protocol to produce a 7 *μ*m thick SiO_2_/Al mask to achieve diamond features beyond 100 *μ*m^14^. To fabricate multilayered devices however, small and precise alignment requirements would be challenging to achieve with shadow masks and the other methods may not be available in all fabrication facilities or best suited to etch depths in the 5–30 *μ*m range. This work aims to provide a simple accessible process to achieve etch depths beyond 10 *μ*m with the microscale precision and alignment benefits of photolithography, and whilst maintaining smooth etched surfaces.

This work investigated the use of an aluminium mask, chosen due to high selectivity and absence of carbide material formation that could cause sample contamination in an Argon/Oxygen Inductively Coupled Plasma (ICP) RIE process for diamond deep etching. Initially, the effect of oxygen content in the Ar/O_2_ plasma on micromasking was examined to determine optimal etching conditions. The process was subsequently evolved into a cyclic Ar/O_2_ and Ar/Cl_2_, reducing micromasking significantly and achieving smooth deep etching down to 10.6 *μ*m.

## Results

### Determination of an optimal Ar/O_2_ gas ratio for ICP RIE and reduced micromasking

**Figure 1:**
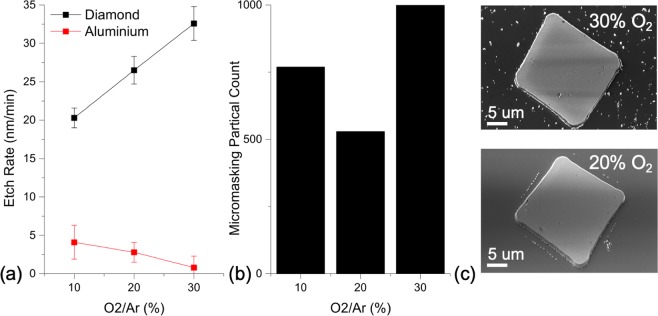
Effect of the Proportion of Oxygen in the Ar/O_2_ Plasma on (**a**) Diamond and Aluminium Etch Rates and (**b**) micromasking Particle Count. As the proportion of oxygen increased, the diamond etch rate also increased whilst the aluminium etch rate reduced. Micromasking was lowest at 20% O_2_. The 30° tilted SEM images (**c**) illustrate the reduction in micromasking observed between 20 and 30% oxygen (410 nm and 489 nm etch depth, respectively). The light grey/white features are protruding, with the square mesa in the centre and micromasking defects around it. The area observed in (**c**) is smaller than the unit area but was chosen to highlight the extent of contrast in micromasking.

The impact of oxygen proportion in the plasma on the aluminium mask, diamond and its surface morphology is presented in Fig. 1. As the plasma gas mix included increasing oxygen levels, from 10% to 30%, the diamond etch rate increased by 60%. The aluminium etch rate on the other hand decreased by 80%. The effect on the etched surface morphology was examined by counting micromasking particles across a unit area and pattern (area dimensions 296 *μ*m × 209 *μ*m) for the different oxygen plasma content, imaged with SEM using particle analysis with ImageJ software^15^. The micromasking particle count reduced at 20% oxygen in argon and was higher in the other cases. Figure 1c illustrates the different observations with significant numbers of small light features - micromasking defects - across the etched surface with 30% O_2_ compared to nearly none with 20% O_2_, and predominantly within close proximity to the square mesa feature.

The observations made in Fig. 1 regarding the increase in diamond etch rate could be explained by the increase in active oxygen species in the plasma, contributing and accelerating removal of material. With regards to the aluminium mask, the etch rate significantly reduced. It was suggested the oxygen, rather than contributing to etching the aluminium, may enable the formation of an oxide layer on the surface of the aluminium mask. Aluminium oxide has a significantly higher tolerance to plasma etching compared to pure aluminium^16^. Furthermore, if the aluminium mask developed higher tolerance to the plasma, mask sputtering and therefore micromasking would likely reduce.

It was noted that further oxidation of the aluminium did not result in continued reduction in micromasking with 30% oxygen in the gas flow. The etch rate reduced by 80% compared to 20% oxygen in the plasma suggesting further oxidation of the mask but micromasking increased. It is postulated that in this case, any sputtered mask particles are more likely to be aluminium oxide and present a very low etch rate resulting in a more marked effect on the surface as the micromasks survive longer in the plasma.

Fixing the gas ratio at 20% oxygen but increasing the ICP power to 200 W maintained observations of minimal micromasing, with the lowest observed micromasking count at 414 particles per unit area. The diamond etch rate increased to 43 nm/min and the aluminium mask etch rate decreased to 2.3 nm/min, with corresponding selectivity of 18.7. The ICP power was not increased further following previous experience by the authors of surface roughening^17^.

**Figure 2:**
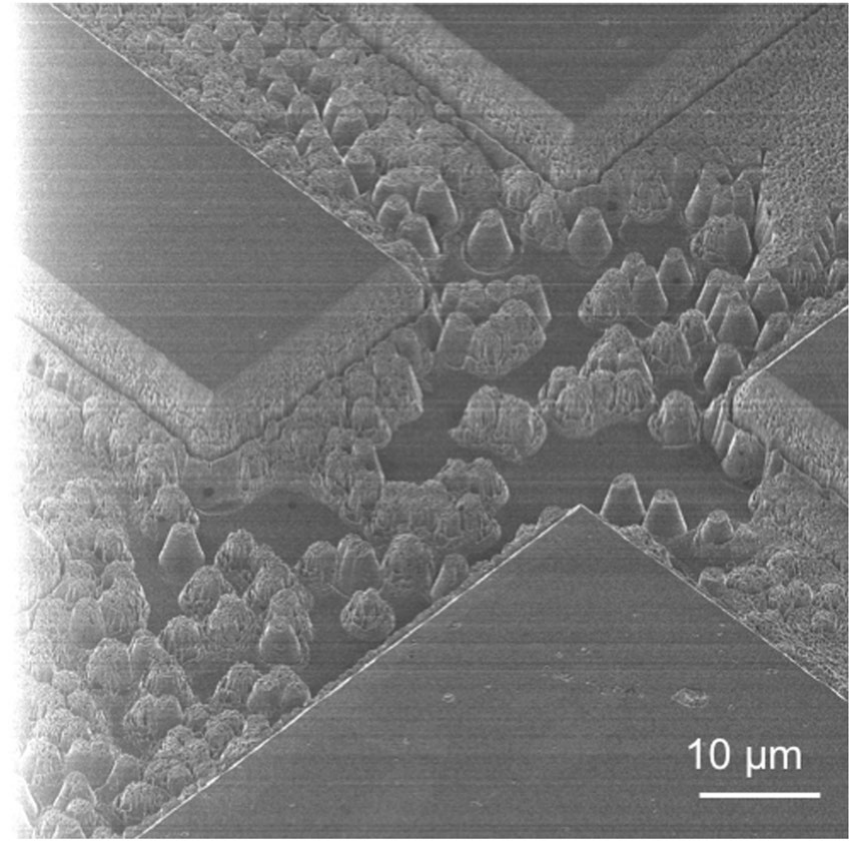
Surface Roughening and Damage Observed after 233 Minute Etch with Ar/O_2_ ICP RIE (HIM). Significant micron-scale pillar-like features were observed across the etched surface. The mesa sidewalls presented a rough surface and trenching at the base.

These ICP RIE parameters were applied to a new sample with the aim of reaching 10 *μ*m etch depth. It was, however, not possible to measure the depth of the etch accurately due to the significant roughening of the etched surface. Features measured at the edge of the chip were 11.5 *μ*m +/− 1 *μ*m. The etched surface, shown in Fig. 2, was covered in micron-scale pillar-like features. The sidewalls of the mesa structures were also significantly roughened, with pits, protrusions and trenching at the base. The wall angle was non-vertical.

### Addition of Ar/Cl_2_ ‘cleaning’ step for near zero micromasking

**Figure 3:**
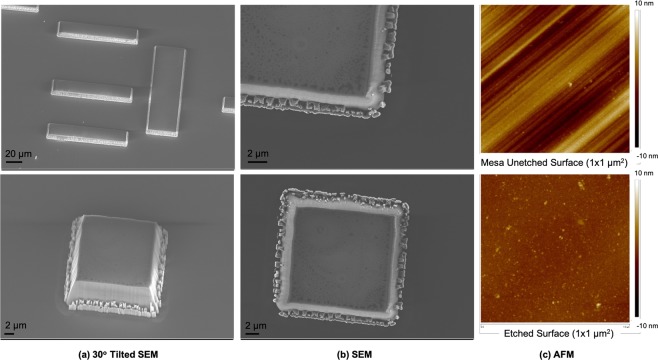
8 *μ*m Deep Etch with Near-Zero Micromasking after Cyclic Ar/O_2_ - Ar/Cl_2_ ICP RIE: (**a**) 30° tilted SEM, (**b**) no tilt SEM and (**c**) AFM scans of the unetched mesa surface with 1.7 nm roughness and etched surface with 0.47 nm roughness. The etched surface was smooth, with micromasking only observed within a 1 micron radius around the base of the mesa. The mesa sidewalls were also smooth with defects observed at the base and without trenching.

**Figure 4:**
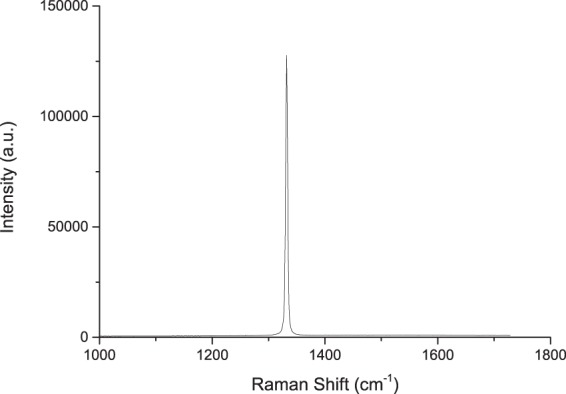
Raman Spectra of the Mesa Sidewall and Surrounding Diamond. The characteristic sharp diamond peak is observed at 1332 cm^−1^ with no sign of graphitic carbon.

The addition of a cyclic chlorine-based plasma step to the process resolved these otherwise very significant micromasking issues. The patterned sample in Fig. 3 presented near-zero micromasking defects across the surface of the samples (154 particles per unit area). The mesa structures were 8 *μ*m in height, with smooth sidewalls. All but 10 micromasking particles were observed within a micron of the features. Compared to previous observations, the micromasking particle count was reduced by a factor of 3.4 for an etch depth approximately 20 times greater. The surface roughness of this sample was measured with AFM to be 0.47 +/− 0.051 nm after the etch, compared to 1.7 +/− 0.49 nm on the unetched mesa surface (Fig. 3c). Figure 4 shows a single peak at 1332 cm^−2^ corresponding to single crystal diamond confirming that the etch process did not produce graphitic carbon on the surface of the etched diamond and sidewalls.

**Figure 5:**
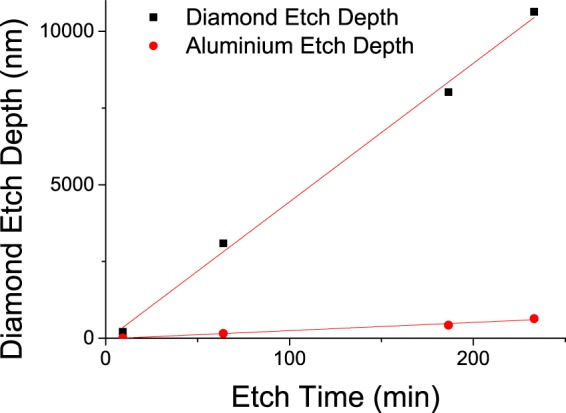
Diamond and Aluminium Mask Etch Depth as a Function of Process Time with Cyclic Ar/O_2_ - Ar/Cl_2_ ICP RIE. The slope of each curve was used as the measure for etch rate, 45.1 nm/min for diamond and 2.67 nm/min for aluminium, for etch depths up to 10.6 *μ*m.

The etch rate for both the diamond and aluminium mask with the cyclic Ar/O_2_ - Ar/Cl_2_ process was determined by processing four different masked chips with different etch times (Fig. 5). The diamond etch rate was 45.1 nm/min with 5% variation and 2.67 nm/min for the aluminium mask, corresponding to a selectivity 16.9.

The etch rate was calculated for features at the centre and on the edges of the 4 × 4 mm^2^ chip with eight *μ*m features (Fig. 3) and showed relatively high uniformity across the sample. The etch rate difference between the centre (43.2 nm/min +/− 0.07 nm standard deviation) and the edge (42.9 nm/min +/− 0.06 nm standard deviation) was marginal. There was also no trenching observed around features further confirming a uniform etch rate. Likewise, there was no variation in the aluminium mask etch rate across the sample. The wall angle was measured at the base of the 8 *μ*m mesas as 81° +/− 3°. Small features in the pattern, 5 *μ*m wide or less, were not fabricated successfully. The mask appeared to erode during the etch process and resulted in features with a significantly narrower top than base.

## Discussion

Diamond poses a significant challenge to achieve high quality deep etching and patterning: the requirement of a metal or oxide mask inevitably introduces micromasking. In this work, the plasma optimisation process was very much focused on addressing the issue of micromasking, without sacrificing etch rate and diamond surface quality to develop the ability to produce deep features.

Initially, an Ar/O_2_ plasma was chosen for its wide availability and known effectiveness in etching diamond smoothly. The argon ions in the plasma provided the main mechanism of physical etching or sputtering. The physical etching was indiscriminate towards defective areas, minimising any etch pits and maintaining a uniformly smooth etched surface. The oxygen ions on the other hand provide a chemical element to the etch mechanism, either by reacting and removing sputtered species or chemically etching carbon atoms from the diamond surface^18^.

Reducing micromasking through plasma optimisation proved insufficient for deep etching as illustrated by the poor quality achieved in the etch in Fig. 2. Additional gas ratios could be examined to ensure an absolute minimum amount of micromasking to O_2_/Ar ratio. *In situ* measurements during plasma processing or vacuum transfer to an X-ray Spectroscopy or X-ray Diffraction system (as aluminium forms a spontaneous native oxide in air) would also enable a better understanding of the effects of the plasma on the aluminium mask. It was however decided to use a cyclic two-step plasma process, with the second plasma specifically aimed at removing aluminium micromasking particles and previously shown to be an effective strategy for SiO_2_ masks on diamond^10^.

Chlorine is a known etchant of aluminium and aluminium oxide so a chlorine-based plasma was chosen for this work^19–21^. Only a small amount of chlorine and a low argon flow was used at lower power than the Ar/O_2_ to avoid reducing selectivity by etching away the mask during the cleaning steps. The frequency of the Ar/Cl_2_ plasma cleaning step was also considered. It needed to be frequent enough to remove micromasks whilst maintaining the use of the cleaning plasma to a minimum to maintain a high mask selectivity.

Figure 3 highlights the clean, smooth, high quality etch achieved down to 8 *μ*m with the Ar/O_2_ - Ar/Cl_2_ protocol. Introducing the cleaning plasma every 2 minutes for 20 seconds successfully removed micromasks across the surface with minimal effect on the selectivity of the process as the diamond and aluminium mask etch rate both increased slightly with the cyclic process. The highest etch depth achieved in this work was 10.6 *μ*m; the process was however not viewed as limited to this depth (only limited in this case by mask thickness and time) and would be expected to deliver similar high quality results at deeper etch depths.

The cyclic Ar/O_2_ - Ar/Cl_2_ process improved sidewall quality with pit-free surfaces and non-vertical angles desirable for applications in electronic devices. Sputtering of the mask onto newly etched sidewalls resulting in temporary protection from the plasma, as suggested by Vargas *et al*., may explain the non-vertical nature of the sidewalls^22^. In the case of the Ar/O_2_ process (Fig. 2), deep pits were observed on the sidewalls. The addition of the cleaning step (Fig. 3) enabled the production of smooth sidewalls, with etch defects limited to a one micron area around the base of the mesa. Increasing the frequency of the ‘cleaning’ plasma step is suggested to produce vertical sidewalls by preventing this temporary protection. The removal of trenching at the base of the mesa was a crucial achievement for electronic device fabrication where a trench could cut through active layers. Further plasma optimisation and characterisation would potentially enable the removal of these etch defects and a better understanding of why micromasking persists in these locations.

The main observed limitation of the Ar/O_2_ - Ar/Cl_2_ cyclic process was the challenge of producing deep features less than five *μ*m in width. In this case, the sidewall masking and angling process referred to above resulted in significantly narrower structures at the top compared to the base of the mesa. These structures may be useful to certain applications like the device structure presented by Iwasaki *et al*.^23^.

## Conclusion

In conclusion, an accessible high quality deep diamond etching process with aluminium mask has been developed by finding an optimal proportion of oxygen in the Ar/O_2_ ICP RIE plasma and introducing a cycling Ar/Cl_2_ micromask cleaning step. The Ar/O_2_ - Ar/Cl_2_ cyclic process enabled the fabrication of up to, but not limited to, 10.6 *μ*m features with smooth etched surfaces and mesa sidewalls, with a 45.1 nm/min etch rate and 16.9 selectivity. Future optimisation will aim to increase etch rate, address the small defects observed at the base of mesas and improve control of sidewall angle and fabrication of features with small widths.

## Methods

Diamond samples used throughout this work were (100) single crystal 4 × 4 mm^2^ chips (Sumitomo and NDT). After a Piranha acid clean and solvent degrease, samples were patterned with bilayer resist (LOR10B and S1818) with a cleanroom UV-photolithography process. Aluminium was thermally evaporated and lifted-off in 1165 Microposit remover to produce the desired mask pattern. The sample was then etched in a Surface Technology Systems ICP SRIE, using a ceramic carrier wafer, back-cooled to 20 °C. Remaining aluminium was removed using an aluminium etchant solution.

Initially the effect of oxygen concentration in the Ar/O_2_ plasma on etch rate and micromasking was examined. The sample was patterned with an aluminium mask covering the whole surface except for a 1 × 1 mm^2^ square with smaller features within. Different areas of the same sample were thus processed under the same plasma conditions but with increasing proportion of oxygen in argon (10, 20 and 30%) by repeating the patterning process and moving the 1 × 1 mm^2^ square across the surface. The argon flow was fixed at 50 sccm, process pressure 5 mTorr, ICP power 100 W and platen power 300 W for 15 minutes. The ICP power was subsequently increased to 200 W with 20% O_2_ in argon for 15 minutes followed by a 233 minute process on a new chip. The aluminium mask thickness was 250–400 nm thick for the 15 minute processes and 1 *μ*m thick for the long etch.

The cyclical two step process was the following: every two minutes of Ar/O_2_ plasma, Ar/Cl_2_ plasma was introduced for 20 seconds with the aim of removing any micromasking effects. The Ar/Cl_2_ plasma was run at 5 mTorr pressure, with ICP power 100 W, platen power 250 W, 15 sccm Ar and 7 sccm Cl_2_. The 2 min Ar/O_2_ - 20 sec Ar/Cl_2_ process was repeated to reach 186.4 minutes (80 times) on a sample with 613 nm thick aluminium mask and 233 minutes (100 times) on a doped epitaxial stack sample with 839 nm thick aluminium mask.

The effect of each etch recipe was characterised with a Dektak profilometer and SEM (Carl Zeiss XB1540 “Cross-beam” FIB microscope and Zeiss ORION NanoFab). The average roughness of the 8 *μ*m mesa sample was measured with a Bruker ICON AFM at 1.5 Hz and 512 lines/scan, averaged for five 1 × 1 *μ*m^2^ scans on the etched surface and three 1 × 1 *μ*m^2^ scans on the unetched mesa surface. The sidewall angle was calculated with trigonometry from the width at the top of the mesa, width at the base of the mesa (both measured with SEM) and height of the mesa (measured with the profilometer) and averaged across three features. The quality of the sidewall was also examined with Raman Spectroscopy (Renishaw Raman Spectrometer).
